# Effect of cardiopulmonary bypass reoxygenation on myocardial dysfunction following pediatric tetralogy of Fallot repair

**DOI:** 10.1186/s12872-021-02033-2

**Published:** 2021-04-26

**Authors:** Ji-nong Yang, Xiao-ming Zhang, Lu-yao Ma, Zhan-jie Lu, Si-qiang Zheng, Al-Wajih Hamzah, Yong-feng Shao, Hong Liu, Gao-li Liu

**Affiliations:** 1 Department of Cardiovascular Surgery, The Affiliated Hospital of Qingdao University, Qingdao, People’s Republic of China 266000; 2 Department of Cardiovascular Surgery, The First Affiliated Hospital of Nanjing Medical University, Nanjing, 210038 People’s Republic of China; 3 School of Public Health Management, Nanjing University of Chinese Medicine, Nanjing, 210029 People’s Republic of China; 4 Department of Cardiovascular Surgery, Teda Cardiovascular Hospital, Chinese Academy of Medical Sciences & Peking Union Medical College, Beijing, 100730 People’s Republic of China

**Keywords:** Hypoxia, Reoxygenation injury, Cardiopulmonary bypass, Tetralogy of Fallot, Perfusate oxygenation

## Abstract

**Background:**

Little is known regarding the effect of cardiopulmonary bypass (CPB) reoxygenation on cardiac function following tetralogy of Fallot repair. We hypothesized that hyperoxic reoxygenation would be more strongly associated with myocardial dysfunction in children with tetralogy of Fallot.

**Methods:**

We investigated the association of perfusate oxygenation (PpO2) associated with myocardial dysfunction among children aged 6–72 months who underwent complete repair of tetralogy of Fallot in 2012–2018. Patients were divided into two groups: lower PpO2 group (≤ 250 mmHg) and higher PpO2 (> 250 mmHg) group based on the highest value of PpO2 during aortic occlusion. The odd ratio (ORs) and 95% confidence intervals (CIs) were estimated by logistic regression models.

**Results:**

This study included 163 patients perfused with lower PpO2 and 213 with higher PpO2, with median age at surgery 23.3 (interquartile range [IQR] 12.5–39.4) months, 164 female (43.6%), and median body mass index 15.59 (IQR 14.3–16.9) kg/m^2^. After adjustment for baseline, clinical and procedural variables, patients with higher PpO2 were associated with higher risk of myocardial dysfunction than those with lower PpO2 (OR 1.770; 95% CI 1.040–3.012, *P* = 0.035). Higher PpO2, lower SpO2, lower pulmonary annular Z-score, and longer CPB time were independent risk factors for myocardial dysfunction.

**Conclusions:**

Association exists between higher PpO2 and myocardial dysfunction risk in patients with tetralogy of Fallot, highlighting the modulation of reoxygenation during aortic occlusion to reduce cardiovascular damage following tetralogy of Fallot repair.

***Trial registration*:**

Clinical Trials. gov number NCT03568357. June 26, 2018

**Supplementary Information:**

The online version contains supplementary material available at 10.1186/s12872-021-02033-2.

## Background

Repair of cyanotic tetralogy of Fallot is increasingly performed in young infants and children [1, 2]. Despite technical improvements, myocardial dysfunction is still challenging that might be mainly attributable in part to chronic hypoxia-reoxygenation injury, which may occur at initiation or during the entire period of cardiopulmonary bypass (CPB) [3–5].

Previous researches have suggested that reoxygenation injury since the onset of hyperoxic supply during CPB would lead to oxide free radical production, lipid peroxidation, and impaired post-bypass contractility, which contributed to severe impairment of functional recovery and myocardial antioxidant reserve capacity after operations [6, 7]. Fortunately, this reoxygenation injury has been attenuated in both animal model and clinical setting by lowing reoxygenation level during initiation of CPB [8]. However, little is known whether high perfusate oxygenation (PpO2) during aortic occlusion is associated with higher risk of myocardial dysfunction following tetralogy of Fallot repair [9, 10].

The primary objective was to estimate the association of PpO2 during aortic occlusion and myocardial dysfunction following tetralogy of Fallot repair overall, by disease-specific pathologies. The secondary objective was to identify the independent risk factors of developing myocardial dysfunction.

## Methods

### Study design and population

This is a retrospective cohort analysis of patients with tetralogy of Fallot between March 2012 and December 2018 at Teda Cardiovascular Hospital. Consecutive patients were included if they underwent complete repair of tetralogy of Fallot with CPB at 6–72 months. We excluded patients who had received extracorporeal membrane oxygenation, intra-aortic balloon pump, ventricular assist device, mechanical ventilatory support, or renal replacement therapy before this current surgery. Additional exclusion criteria were incomplete PpO2 records and missing outcome data for identification of postoperative myocardial dysfunction.

The Institutional Review Board of Teda Cardiovascular Hospital approved the study. This study followed the Declaration of Helsinki and the ethical standards of the responsible committee on human experimentation. Informed consent was waived for this retrospective study. This study was registered with Clinical Trials. gov number NCT03568357.

### Study exposure

Patients were totally heparinized, and an activated clotting time of more than 480 secs was confirmed before the initiation and duration of CPB. Pump flow was settled at approximately 2.8 L/min/m^2^ at the outset of CPB and subsequently adjusted according to patient’s core temperature (usually a 20% decrease for core temperatures between 30 and 34 °C and an additional 10% decrease for core temperatures < 30 °C). The hemodilution level was decreased to 25–30% during hypothermic CPB according to the routine protocol of our institution [11].

We hypnotized that exposure to hyperoxic CPB would lead to reoxygenation injury in cyanotic patients, so we used the highest PpO2 during aortic occlusion for analysis. Patients were divided into two groups: lower PpO2 group (< 250 mmHg) and higher PpO2 group (> 250 mmHg). PpO2 was available from online blood gas analyzers and confirmed by an independent manual check of extracorporeal circulation records by blinded trained staff.

### Outcome

Primary outcome was myocardial dysfunction, which was confirmed if any of the following is true:

1. need for higher doses of vasoactive drug to maintain blood pressure in normal range (vasoactive-inotropic score [VIS] of greater than 15 points [12]);
2. decrease in blood pressure < 5th percentile for age or systolic blood pressure < 2 SD below normal for age [13];
3. two of the following: unexplained metabolic acidosis, oliguria, prolonged capillary refill > 5 s, core to peripheral temperature gap > 3 °C [13]; OR
4. Postoperative use of renal replacement therapy, extracorporeal membrane oxygenation, intra-aortic balloon pump, or ventricular assist device [14].

Myocardial dysfunctions were determined throughout postoperative 7 days, hospital discharge, or death, whichever occurred first. Secondary outcome included inotropic score, ICU stay, mechanical ventilation support time, postoperative stay, and hospital cost, as well as the rate of postoperative acute lung injury [15] and systemic inflammatory response syndrome [16].

### Study covariates

Baseline clinical characteristics included age at surgery, sex, body mass index (BMI), hematocrit, percutaneous saturation (SpO_2_), left ventricular end-diastolic volume (LVEDV), and NYHA classification. Disease-specific pathologies included McGoon ratio, pulmonary annular Z-score, and aortopulmonary collateral arteries (APCAS). Procedural characteristics included cardioplegia types, transannular patch, tricuspid valve detachment, CPB time, and intraoperative blood transfusion, as well as immediately postoperative central venous pressure (post-CVP) and transpulmonary gradient (post-TPG).

### Statistical analysis

Continuous data were presented as mean (SD) or median (IQRs) and compared using a t-test or Kruskal–Wallis testing depending on distributed characteristics, and categorical data were reported as percentages (%) and compared using χ2 or Fisher’s exact testing. The odd ratio (ORs) and 95% confidence intervals (CIs) were estimated by logistic regression models. Baseline adjustments included age at surgery, sex, BMI, hematocrit, SpO2, LVEDV, and NYHA classification. Clinical adjustments included McGoon ratio, pulmonary annular Z-score, and APCAS. Procedural adjustments included cardioplegia types, transannular patch, tricuspid valve detachment, CPB time, intraoperative blood transfusion, post-CVP, and post-TPG.

We conducted stratified risk analyses by disease-specific characteristics (pulmonary annular Z-score, McGoon ratio, and APCAS). Heterogeneity of these stratified estimates was evaluated using the likelihood ratio test of the interaction terms between PpO2 and each covariate.

All covariates showing relative strong associations (*P* value < 0.1) with myocardial dysfunction in univariate analysis were modelled together to investigate independent risk factors of myocardial dysfunction using multivariate logistic regression. All statistical analysis were performed using Stata version 14 (Stata Corp, College Station, TX, USA) and R software (version 3.2.0). *P* values of less than 0.05 were considered statistically significant.

All methods were carried out in accordance with relevant guidelines and regulations.

## Results

### Patient characteristics

Our study included 163 patients perfused with lower PpO_2_ and 213 patients with higher PpO_2_. Regarding baseline, clinical, and procedural characteristics, patients with higher PpO_2_ had lower proportion of male (*P* < 0.0001), lower SpO_2_ (*P* = 0.004), lower LVEDV index (*P* = 0.014), lower pulmonary annular Z-score (*P* = 0.014), larger blood transfusion (*P* = 0.017) and longer CPB time (*P* = 0.013) (Table 1). Patients perfused with lower and higher PpO_2_ had similar distribution on age at surgery, hematocrit, BMI, proportion of NYHA class, McGoon ratio, APCAS, cardioplegia types, transannular patch, tricuspid valve detachment, post-CVP, and post-TPG (all *P* > 0.05).

**Table 1:** Study population and participant characteristics

	Total	Lower PpO2 (163)	Higher PpO2 (213)	*P* value
*Baseline clinical variables*				
Age at surgery, month	23.3 (12.5–39.4)	22.77 (12.9–39.4)	24.60 (12.4–38.8)	0.975
Sex, %				< 0.001
Female	164 (43.6%)	55 (33.7%)	109 (51.2%)	
Male	212 (56.4%)	108 (66.3%)	104 (48.8%)	
Body mass index, %	15.59 (14.3–16.9)	15.60 (14.3–16.8)	15.58 (14.3–16.8)	0.974
Hematocrit, %	41.7 (37.4–48.5)	40.70 (37.0–47.0)	42.90 (38.1–49.6)	0.141
SpO2, %	84.0 (77.7–93.0)	86.00 (78.5–95.0)	82.0 (77.0–92.0)	0.004
NYHA class				0.670
I–II	353 (93.9%)	152 (93.3%)	201 (94.4%)	
III–IV	23 (6.1%)	11 (6.8%)	12 (5.6%)	
LVEDV index, ml/m^2^	27.02 (19.96–35.66)	29.00 (22.59–36.88)	25.84 (18.84–34.63)	0.014
Pulmonary annular z-score				
< − 4.0, %	101 (26.9%)	50 (30.7%)	51 (23.9%)	0.048
− 2.0 to − 4.0, %	117 (31.1%)	40 (24.5%)	77 (36.2%)	
> − 2.0, %	158 (42.0%)	73 (44.8%)	85 (39.9%)	
McGoon index				
≤ 1.5, %	138 (37.1%)	54 (33.5%)	84 (39.8%)	0.215
> 1.5, %	234 (62.9%)	107 (66.5%)	127 (60.2%)	
APCAS, %				0.828
None-minimal (untreated)	187 (49.9%)	84 (51.5%)	103 (48.6%)	
Minor (untreated)	79 (21.1%)	34 (20.9%)	45 (21.2%)	
Major (treated)	109 (29.1%)	45 (27.6%)	64 (30.2%)	
*Procedural variables*				
Cardioplegia, %				0.500
4:1 (Buckberg)	129 (34.3%)	59 (36.2%)	70 (32.9%)	
1:4 (del Nido)	247 (65.7%)	104 (63.8%)	143 (67.1%)	
*Transannular patch, %*				0.880
Absence	165 (44.0%)	71 (43.6%)	94 (44.3%)	
Presence	210 (56.0%)	92 (56.4%)	118 (55.7%)	
*Tricuspid valve detachment, %*				0.461
Absence	304 (80.8%)	129 (79.1%)	175 (82.2%)	
Presence	72 (19.2%)	34 (20.9%)	38 (17.8%)	
*Blood transfusion, %*				0.017
< 10 ml per kg	60 (16.0%)	27 (16.6%)	33 (15.5%)	
10–30 ml per kg	121 (32.2%)	56 (34.4%)	65 (30.5%)	
> 30 ml per kg	195 (51.8%)	80 (49.0%)	115 (54.0%)	
Post-CVP, %				0.530
5–10 cmH_2_O	274 (72.9%)	123 (75.5%)	151 (71.0%)	
< 5 cmH_2_O	30 (8.0%)	13 (8.0%)	17 (7.9%)	
> 10 cmH_2_O	72 (19.2%)	27 (16.5%)	45 (21.1%)	
Post-TPG, %				0.643
< 15 mmHg	99 (26.3%)	46 (28.2%)	53 (24.9%)	
15–30 mmHg	160 (42.6%)	70 (42.9%)	90 (42.2%)	
> 30 mmHg	117 (31.1%)	47 (28.8%)	70 (32.9%)	
CPB time, min	123.5(90.0–158.5)	116.0 (88.5–143.0)	131.0 (92.0–169.0)	0.013

Continuous data are presented as median (IQR) and dichotomous data are presented as counts (%). PpO2 = perfusate oxygenation; SpO2 = percutaneous oxyhemoglobin saturation; LVEDV = left ventricular end-diastolic volume; APCAS = aortopulmonary collateral arteries; CPB = cardiopulmonary bypass; post-CVP = immediately postoperative central venous pressure; post-TPG = immediately postoperative transpulmonary gradient

### Hospital outcomes

In relative to those with lower PpO_2_, patients with higher PpO_2_ were associated with significantly higher maximum inotropic score, longer ICU stay, longer mechanical ventilation support, longer postoperative stay, and more hospital cost (all *P* < 0.05) in Table 2. Besides, patients with higher PpO_2_ had higher percentage of acute lung injury (30.67% vs 41.51%; *P* = 0.031) and systemic inflammatory response syndrome (5.37% vs 11.86%; *P* = 0.038) than those with lower PpO_2_ (online Table 1).

**Table 2:** Odds ratios for cardiovascular dysfunction by categorical perfusate oxygenation

	Odds ratio (95% CI )	*P* value
Crude model		0.032
≤ 250 mmHg	1.000 (ref)	
> 250 mmHg	1.753 (1.048, 2.933)	
Adjusted for baseline covariates*		0.042
≤ 250 mmHg	1.000 (ref)	
> 250 mmHg	1.746 (1.019, 2.991)	
Adjusted for baseline and clinical covariates^#^		0.044
≤ 250 mmHg	1.000 (ref)	
> 250 mmHg	1.701 (1.014, 2.855)	
Adjusted for baseline, clinical and procedural covariates^$^		0.035
≤ 250 mmHg	1.000 (ref)	
> 250 mmHg	1.770 (1.040, 3.012)	

*Baseline adjustments for age at surgery, sex, body mass index (BMI), hematocrit, SpO2, left ventricular end-diastolic volume (LVEDV), and NYHA classification

^#^Clinical adjustments for added clinical factors including McGoon ratio, pulmonary annular Z-score, and aortopulmonary collateral arteries (APCAS)

^$^Procedural adjustments for added factors including cardioplegia types, transannular patch, tricuspid valve detachment, cardiopulmonary bypass (CPB) time, and intraoperative blood transfusion, immediately postoperative central venous pressure (post-CVP), and transpulmonary gradient (post-TPG)

### Association of Ppo2 with myocardial dysfunction

In unadjusted logistic model, patients with higher PpO_2_ were associated with higher risk of myocardial dysfunction relative to those with lower PpO_2_ (OR 1.753; 95% CI 1.048–2.933; *P* = 0.032). Adjusted for baseline and clinical variables, there still exists significant association between categorical Ppo2 and risk of myocardial dysfunction (OR 1.701; 95% CI 1.014–2.855, *P* = 0.044). After adjustment for baseline, clinical and procedural variables, patients with higher PpO_2_ were also associated with higher risk of myocardial dysfunction than those with lower PpO_2_ (OR 1.770; 95% CI 1.040–3.012, *P* = 0.035) (Table 2).

### Subgroup analysis for disease-specific pathologies

**Fig. 1:**
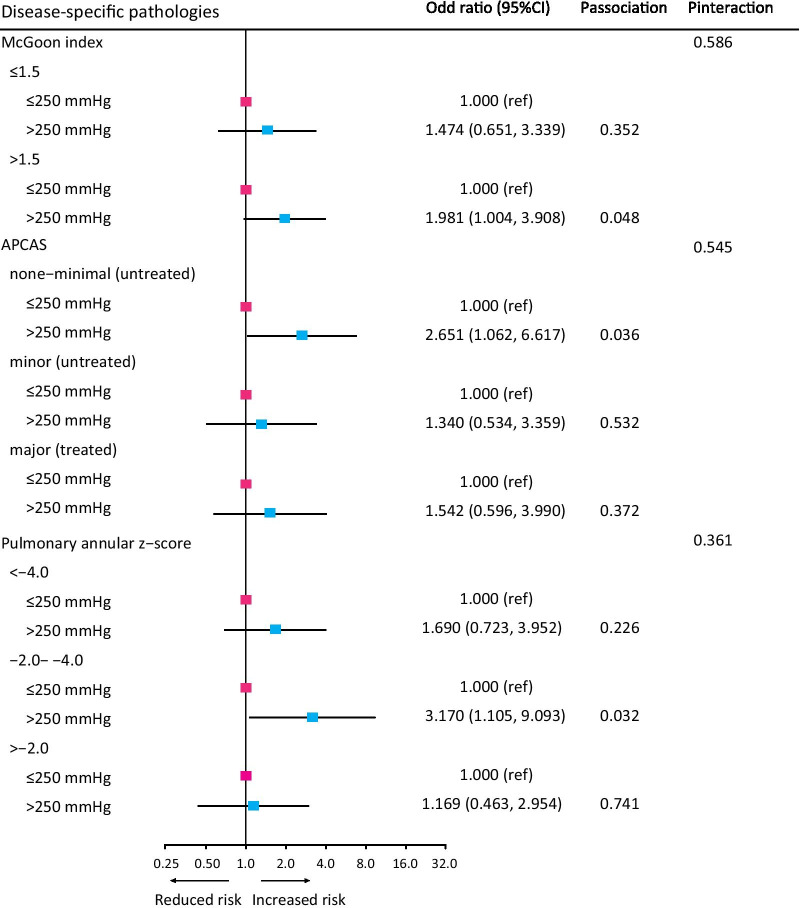
Odd ratio for myocardial dysfunction by disease-specific pathologies. PpO2 = perfusate oxygenation; APCAS = aortopulmonary collateral arteries; CI = confidence Interval

The association between PpO_2_ and risk of myocardial dysfunction by disease-specific pathologies were summarized in Fig. 1. Despite no statistical evidence for interaction (all P interaction > 0.05), there was some indications that patients perfused with higher PpO_2_ were associated with a trend towards increased risk of myocardial dysfunction compared with those with lower PpO_2_ in disease-specific subgroups.

### Independent risk factors of myocardial dysfunction

All significant variables (*P* < 0.10) in the univariable analysis were entered into the multivariable logistic regression model. The multivariate analysis indicated that categorical PpO_2_, lower SpO_2_, lower pulmonary annular Z-score, and longer CPB time were independent risk factors for myocardial dysfunction (Table 3).

**Table 3:** Multivariable logistic regression analysis for cardiovascular dysfunction risk

	Odds ratio (95% CI)	*P* value
*Categorical PpO*_*2*_*, %*		
Lower PpO2	1.000 (ref)	
Higher PpO2	1.858 (1.078, 3.202)	0.026
*Clinical covariates*		
Sex, %		0.667
Female	1.000 (ref)	
Male	0.886 (0.510, 1.539)	
SpO_2_ (per % increase)	0.954 (0.927, 0.983)	0.002
LVEDV index, ml/m^2^	1.005 (0.981, 1.030)	0.668
Pulmonary annular Z-score, %		
< − 4.0	1.000 (ref)	
− 2.0 to − 4.0	0.747 (0.395, 1.415)	0.372
> − 2.0	0.451 (0.220, 0.924)	0.030
McGoon index, %		0.341
≤ 1.5	1.000 (ref)	
> 1.5	1.325 (0.742, 2.366)	
Blood transfusion, %		
None (< 10 ml per kg)	1.000 (ref)	
Minor (10–30 ml per kg)	1.011 (0.481, 2.125)	0.978
Major (> 30 ml per kg)	1.015 (0.455, 2.264)	0.972
CPB time (per min increase)	1.009 (1.004, 1.014)	0.0002

PpO_2_ = perfusate oxygenation; SpO_2_ = percutaneous oxyhemoglobin saturation; LVEDV = left ventricular end-diastolic volume; CPB = cardiopulmonary bypass. CI = confidence interval

## Discussion

Our findings show that significant association of higher PpO2 during aortic occlusion with myocardial dysfunction risk after adjustment for potential variables. There were some indications that patients perfused with higher PpO_2_ were associated with a trend towards increased risk of myocardial dysfunction compared with those with lower PpO_2_ in disease-specific subgroups. Higher PpO2, lower SpO2, lower pulmonary annular Z-score, and longer CPB time were independent risk factors for myocardial dysfunction.

### Interpretation and implications

Different from previous studies regarding the reoxygenation during initiation of CPB [5–8, 17], we have an exclusive focus on the reoxygenation during aortic occlusion. Our findings showed that patients perfused with higher PpO2 were associated with higher risk of myocardial dysfunction relative to those perfused with lower PpO2mmHg despite multivariable adjustments for clinical and procedural variables, which is consistent with their previous results, which provided evidence verifying impaired cardiac function and of biochemical parameters closely correlating with poor surgical outcome attributable to reoxygenation injury [3, 4, 18]. Thus, it highlights the therapeutic modalities of reoxygenation not only during initial CPB but also during aortic occlusion process in the management of oxygenation in tetralogy of Fallot repair [19].

Myocardial dysfunction is multifactorial in etiology, including myocardial depression following bypass, altered cardiovascular loading conditions, and inflammation driving a hypermetabolic state, which particularly associated with procedural factors in addition to patient baseline clinical characteristics [20, 21]. Not surprisingly, our multivariate analysis identified lower SpO2, lower pulmonary annular Z-score, and longer CPB time were independent risk factors of myocardial dysfunction. Consequently, understanding and accurately estimating perioperative risk by accounting for the intrinsic risk of surgical procedures and patient clinical characteristics will lead to a timely and accurate use of hemodynamic monitoring for a better identification and management of patients as risk for or in a state of myocardial dysfunction [22, 23].

It is interesting that the presence of transannular patch did not warrant retention in our multivariable model in light of previous work demonstrating that in patients undergoing tetralogy of Fallot repair transannular patch is associated with increased risk of postoperative low cardiac output syndrome [24]. This is likely due to the fact that the presence of transannular patch was considered as a binary variable in this analysis and the specific RVOT-related procedures (e.g. parietal muscle resection, infundibular outflow patch, valve-sparing surgery, transannular patch, or pulmonary patch) was not considered when predicting myocardial dysfunction in this present study. It is also likely that these infants and children who underwent transannular patch usually had high clinical risk more frequently, such as lower preoperative SpO2 which was included in the prediction model as an impendent risk factor, than those who did not underwent transannular patch [25].

There are certain limitations to this study. While we used a consensus-based definition for myocardial dysfunction, the selection of threshold of vasoactive-inotropic score is neither uniform nor specific, and we cannot rule out some misclassification. Despite multivariable adjustments for measured factors for this analysis, the residual bias from unmeasured factors and effect-modifying potentials cannot be completely excluded.

## Conclusions

Our findings showed higher Ppo2 during aortic occlusion was associated with risk of myocardial dysfunction, highlighting the modulation of reoxygenation during aortic occlusion to reduce cardiovascular damage in patients with tetralogy of Fallot. Also, higher PpO2, lower SpO2, lower pulmonary annular Z-score, and longer CPB time were independent risk factors for myocardial dysfunction. However, the unmeasured factors with confounding and effect-modifying potentials affecting myocardial dysfunction cannot be excluded.

## Supplementary Information

**Additional file 1: Online Table 1**. Hospital outcome.

## Data Availability

The datasets used and/or analysed during the current study are available from the corresponding author on reasonable request.
