# Supplementary material for: Effect of cardiopulmonary bypass reoxygenation on myocardial dysfunction following pediatric tetralogy of Fallot repair
Source: BMC Cardiovasc Disord. 2021 Apr 26;21:210. doi: 10.1186/s12872-021-02033-2 (PMC8074462; doi:10.1186/s12872-021-02033-2)
Supplement: Supplementary file 1 — Additional file 1: Online Table 1. Hospital outcome. [file 12872_2021_2033_MOESM1_ESM.docx]

**Online Table 1 Hospital outcome**

|  | **Total** | **Lower PpO2 (163)** | **Higher PpO2 (213)** | **P valve** |
| --- | --- | --- | --- | --- |
| Maximum VIS*, point | 9.0 (6.0-13.5) | 8.70 (5.0-12.5) | 10.00 (6.0-14.0) | 0.046 |
| ICU stay, hrs | 69.0 (44.0-139.2) | 65.00 (43.0-102.0) | 71.00 (45.0-151.0) | 0.010 |
| MV time, hrs | 22.0 (12.8-69.0) | 21.00 (7.5-45.0) | 24.0 (18.0-92.0) | 0.002 |
| MV time >48 hrs, % | 116 (30.9%) | 37 (22.7%) | 79 (37.1%) | 0.003 |
| MV time >72 hrs, % | 102 (27.1%) | 34 (20.9%) | 68 (31.9%) | 0.017 |
| Acute lung injury, % | 138 (36.8%) | 50 (30.7%) | 88 (41.5%) | 0.031 |
| SIRS, % | 31 (9.0%) | 8 (5.4%) | 23 (11.9%) | 0.038 |
| Hospital cost, ¥ | 43840 (36007-58844) | 41914 (36146-50451) | 45453 (35823-62259) | 0.026 |
| Hospital stay, days | 9.0 (7.0-13.0) | 8.0 (7.0-11.0) | 9.00 (7.0-14.0) | 0.011 |
| In-hospital mortality, % | 6 (1.6%) | 1 (0.6%) | 5 (2.4%) | 0.240 |

Continuous data are presented as median (IQR) and dichotomous data are presented as counts (%). IS=inotropic score; VIS=vasoactive-inotropic score; ICU= intensive care unit; MV=mechanical ventilation; SIRS= Systemic Inflammatory Response Syndrome.

*VIS was calculated daily as dopamine dose (μg/kg/min) + dobutamine dose (μg/kg/min) + 100 × epinephrine dose (μg/kg/min) + 10 × milrinone dose (μg/kg/min) + 10,000 × vasopressin dose (units/kg/min) + 100 × norepinephrine dose (μg/kg/min).
